# Determination of Reference Intervals for Selected Liver Biomarkers With Focus on Pre‐ and Postanalytical Factors in Lactating Dairy Cattle

**DOI:** 10.1111/vcp.70043

**Published:** 2025-10-11

**Authors:** Anna Theile, Merle Hardekopf, Marion Schmicke

**Affiliations:** ^1^ Veterinary Endocrinology and Laboratory Diagnostics Clinic for Cattle, University of Veterinary Medicine Hannover Hannover Germany

**Keywords:** buiatrics, clinical chemistry, Holstein Friesian, photometry

## Abstract

**Background:**

Measurement of liver enzymes and metabolites for both clinically healthy and sick cattle is a routine part of dairy herd management. Reference intervals (RIs) are influenced by many variables, including pregnancy, breeding, and geographical variables, and can shift over time. Few previous studies have addressed the specific RIs of dairy cows, and none have specifically addressed the RIs of German Holstein Friesian cows.

**Objective:**

The aim was to determine the RIs of German Holstein Friesian cows considering variables such as age, parity, milk yield, body condition score, days in milk, and pregnancy. Additionally, this study aimed to evaluate the effects of anticoagulant use on the RIs.

**Methods:**

Serum, lithium heparin, and EDTA plasma samples from 131 lactating, apparently healthy Holstein Friesian cows from 10 dairy farms were collected. The levels of glutamate dehydrogenase (GLDH), γ‐glutamyltransferase (GGT), aspartate aminotransferase (AST), alanine aminotransferase (ALT), alkaline phosphatase (ALP), creatine kinase (CK), cholesterol, nonesterified fatty acids (NEFAs), and total bilirubin were measured, and new RIs were determined.

**Results:**

The following RIs were determined: beta‐hydroxybutyrate (BHB): 0.25–1.00 mmol/L; ALP: 71.1–258.9 U/L; GLDH: 9.1–121.0 U/L; CK: 65.4–257.2 U/L; GGT_pregnant_: 8.7–65.3 U/L; GGT_not pregnant_: 12.3–48.6 U/L; NEFAs_> 42 Day in Milk_: 65.4–308.7 μmol/L; ALT: 14.0–34.5 U/L; cholesterol: 2.7–7.5 mmol/L; AST_pregnant_: 76.6–279.7 U/L; AST_not pregnant_: 67.1–187.1 U/L; total bilirubin: 0.9–5.2 μmol/L.

**Conclusion:**

New and more precise RIs for cattle could help veterinarians detect hepatocyte damage with minor enzyme leakage and liver stress at an early stage.

## 1 Introduction

The liver plays a central role in the metabolism of dairy cattle. Therefore, measuring liver enzyme activity and metabolites is important for herd health management on dairy farms [1]. To interpret variables that are measured via laboratory techniques, reference intervals (RIs) that are specifically suitable for high‐yielding dairy cows should be used. Many physiological variables, for example, age, parity, pregnancy, and stage of lactation, as well as geographical variables, have an impact on RIs; these variables should be considered when establishing RIs [2, 3]. Moreover, the choice of anticoagulant may also affect the RIs of certain analytes in cattle [4]. In addition to these factors, another very important factor is breeding. As breeding for high performance in dairy cows in recent decades may have led to a shift in RIs, RIs older than 10 years should no longer be used [5]. However, few recent studies have focused on specific RIs in cattle [6, 7, 8]. Moretti et al. [6] calculated RIs for chemical biomarkers at the beginning of the lactation period, from Day 1 to Day 30 [6]. Two other studies have reported several RIs for factors such as parity, days in milk (DIM), and season [7, 8]. Italian Holstein Friesian cows were used in all three studies. To our knowledge, no studies have reported the effects of anticoagulant use on the establishment of RIs in Holstein Friesian dairy cows.

RIs for lactating German Holstein Friesian dairy cows, considering physiological and preanalytical factors such as anticoagulants, have not been widely reported. Therefore, this study aimed to establish improved and precise RIs for Holstein Friesian cattle and to investigate the advantages of separate RIs in terms of age, parity, milk yield, body condition score (BCS), DIM, and pregnancy status. The effects of different anticoagulants on the RIs were also evaluated. These RIs could help veterinarians detect minor enzyme leakage due to, for example, ketosis, increased reactive oxygen species levels, or fatty liver disease, and even early stages of liver stress after parturition.

## 2 Materials and Methods

### 2.1 Animal Selection

Sample collection took place between December 2023 and January 2024. A total of 131 lactating Holstein Friesian cows from 10 dairy farms in northern Germany were sampled. The selected sample size of *n* = 131 was calculated by nonparametric case number calculation using the program NCSS‐Pass, version 23.0.1 (NCSS LLC, Kaysville, UT, USA).

The results from 115 routine samples were used as the basis for the calculation. The calculation with NCSS‐PASS is based on the 95% level of reference intervals. The confidence level was set at 90%, and the margin of error was 15% [9]. The calculation revealed 115 samples; however, to meet the recommendations of the American Society of Veterinary Clinical Pathology (ASVCP) guidelines and because not all analyzed clinical chemistry values were used for the power analyses, the sample size was increased by approximately 4% [10]. Only farms that met the specified selection criteria were included in the study (Table 1). The samples used were leftover samples from an animal experiment approved by the Lower Saxony State Office for Consumer Protection and Food Safety (animal experimental approval no. 33.33‐42502‐04‐23‐00446). For each farm, 13–14 cattle were randomly selected by using the RANDBETWEEN function in Microsoft Excel 2016 (Microsoft Corporation, Redmond, WA, USA). All animals were lactating, and dry cows were not included. A standardized general examination was carried out on each animal before sampling, and only apparently healthy cows were included in the study (Table 1).

**TABLE 1:** Criteria for farms and cows included in the study.

Evaluation criteria	Study inclusion criteria
Farm	
Feeding	TMR/partial TMR
Feeding system	Feed mixer wagon, automatic feeding systems
Ration design	Feeding based on an adjusted ration calculation
Husbandry	Compliance with Council Directive 98/58/EC
Husbandry system	Loose‐housing barn, loose‐housing barn with grazing
Milk yield	On average, > 8500 kg/cow/year
Pregnancy examination	Pregnancy test
Fertility data recording	Recording of insemination and pregnancy data
Cattle	
Breed	Holstein Friesian
Gender	Female
Lactation	Lactating
Body temperature	38.0°C–39.0°C
Respiratory rate	20–40 bpm, without breathing noises
Heart rate	50–95 bpm, without cardiac murmurs
Behavior	Calm and attentive
Posture	All four limbs loaded evenly with head held freely with a straight back
BCS [11]	2.5–4.0
Condition	Good standard of care
Habitus	The habitus of a healthy animal

Abbreviations: BCS, body condition score; TMR, total mixed ratio.

### 2.2 Blood Sampling and Analytical Methods

Blood was taken from the external jugular vein and collected in a serum tube (S‐Monovette 9 mL Z, Sarstedt AG & Co. KG, Nümbrecht, Germany), a lithium heparin tube (S‐Monovette LH, Sarstedt AG & Co. KG), or an EDTA tube (Monovette 9 mL K3E, Sarstedt AG & Co. KG). Before the vessel was punctured using a Strauss cannula (2.0*43 mm, 14 G, Veterinär medizinische Produkte GmbH, Hückelhoven, Germany), the puncture site was disinfected with 70% ethanol. Serum and lithium heparin samples were taken from the vein using a blood collection plug‐in system with multiple adaptors (Sarstedt AG & Co. KG). The order of collection was based on the guidelines of the National Committee for Clinical Laboratory Standards (NCCLS), starting with the sample tube without additive, followed by lithium heparin, and finally the EDTA additive. The samples were collected from all animals between 09:00 and 12:00.

After blood sampling, the EDTA and lithium heparin samples were gently swirled 10 times and stored together with the serum samples for 60 min at room temperature in an upright position. Furthermore, the blood samples were centrifuged (1500 *g*, room temperature, 15 min, Hettich centrifuge, Andreas Hettich GmbH & Co. KG, Tuttlingen, Germany). Afterward, the serum and plasma were transferred into tubes (5 mL, Sarstedt AG & Co. KG) and sealed with stoppers (press‐in stoppers, Sarstedt AG & Co. KG). Prior to analysis, the sample material was stored at −20°C ± 2°C.

All analyses were performed using a Pentra C400 ISE Clinical Chemistry Analyzer (HORIBA Europe GmbH, Oberursel, Germany) at the Clinical‐Endocrinology Laboratory, Clinic for Cattle, University of Veterinary Medicine Hannover, Germany. Before measurement, the plasma and serum were thawed and mixed thoroughly. The measurements and analytical methods for the three sample materials are shown in Table 2. Only commercially available test kits were used, following the manufacturer's instructions.

**TABLE 2:** Test kits and methods used, with measurement ranges and calculated coefficients of variation (CVs) for interassay imprecision, observed total error (TE_obs_) and allowable total error (TE_a_) [12].

Variable	Test kit	Method	Measurement range	Interassay imprecision CV (%)	TE_obs_ (%)	TE_a_ (%)
BHB	RANBUT D‐3‐Hydroxybutyrate, Randox Laboratories Ltd., Crumlin, Northern Ireland	NADH oxidation of D‐3‐hydroxybutyrate	0.1–5.75 mmol/L	9.1	28.6	NL
ALP	Alkalische Phosphatase, Labor + Technik, Eberhard Lehmann GmbH, Berlin, Germany	Nitrophenylphosphate reaction	8–1825 U/L	6.1	22.4	25.0
GLDH	GLDH, Roche Deutschland Holding GmbH, Grenzach‐Wyhlen, Germany	NADH oxidation with alpha‐ketoglutarate	1–80 U/L	3.2	17.5	30.0
CK	CK NAC ekt., Labor + Technik, Eberhard Lehmann GmbH, Berlin, Germany	N‐acetyl‐cystein‐(NAC)‐activated	2–2300 U/L	1.6	4.7	30.0
GGT	GAMMA GT, DIALAB Produktion und Vertrieb von chem.‐tech. Produkten und Laborinstrumenten GmbH, Wiener Neudorf, Austria	Szasz method	2–248 U/L	1.7	10.7	20.0
NEFA	NEFA‐HR (2), Wako Chemicals GmbH, Neuss, Germany	Acyl synthase, oxidase, peroxidase	10–4000 μmol/L	6.6	15.5	NL
ALT	ABX Pentra ALT CP, Horiba ABX SAS, Montpellier, France	NADH oxidation without pyridoxal phosphate	4.0–600.0 U/L	6.4	13.0	25.0
Cholesterol	Cholesterin, Labor + Technik, Eberhard Lehmann GmbH, Berlin, Germany	Cholesterol oxidase, esterase & peroxidase	0.5–19.5 mmol/L	4.8	13.0	20.0
AST	ASAT GOT, Labor + Technik, Eberhard Lehmann GmbH, Berlin, Germany	NADH oxidation without pyridoxal phosphate	2–300 U/L	8.8	26.7	30.0
Total bilirubin	Bilirubin, Labor + Technik, Eberhard Lehmann GmbH, Berlin, Germany	Jendrassik‐Grof method	1.7–425.0 μmol/L	14.1	15.5	25.0

Abbreviations: ALP, alkaline phosphatase; ALT, alanine aminotransferase; AST, aspartate aminotransferase; BHB, beta‐hydroxybutyrate; CK, creatine kinase; GGT, gamma‐glutamyltransferase; GLDH, glutamate dehydrogenase; NEFA, nonesterified fatty acids; NL, not listed.

Quality control tests were performed for each measurement using a human control kit and a bovine pool every day before the samples were analyzed. The kits used for the analysis were calibrated according to the manufacturer's instructions. An interassay imprecision experiment with one measurement taken per day for 20 days from the bovine pool and human control kit was performed, and the coefficient of variation was determined. The observed total error (TE_obs_) was calculated for the human control kit using the formula TE_obs_ = 2CV + Bias% (Table 2) [13]. The TE_obs_ was compared with the allowable total error (TE_a_) [12]. The performance was assumed to be acceptable if TE_obs_ was less than TE_a_.

### 2.3 Statistical Analyses

The data were analyzed using R Studio (R version 4.3.0, Posit PBC, Boston, MA, USA) and GraphPad Prism (version 10, GraphPad Software Inc., Boston, MA, USA).

Possible correlations between age, number of lactations, DIM, milk yield, and BCS and the levels of the investigated variables in serum were analyzed using Pearson's correlation or Spearman's correlation for non‐normally distributed data. The variables investigated were measured only in serum, as this is the recommended standard material for clinical chemistry [14]. This regression analysis included the calculation of the coefficient of determination and the establishment of a regression line and equation and was also performed using serum measurements. The effect of pregnancy on serum samples was analyzed using the Mann–Whitney *U* test or unpaired *t* test, depending on whether the data were normally distributed. The effect of sample material on the measurements was statistically analyzed using repeated‐measures ANOVA or the Friedman test for non‐normally distributed data. The *p* value was adjusted using the Bonferroni method, and *p* values ≤ 0.01 were considered significant.

RIs were calculated in accordance with the ASVCP guidelines [10]. First, a histogram was generated to provide an overview of the data and to identify outliers. Visually identified outliers were checked for errors in transcription, preanalysis, or analysis. A correction or elimination was subsequently made. These datasets were subsequently tested for a normal distribution using the Shapiro–Wilk test and a quantile–quantile plot and for symmetry using the Kolmogorov–Smirnov test. If the data were not normally distributed, the dataset was transformed using the Box–Cox method, and the data were then retested. The outliers were checked using Dixon outlier identification and removed if necessary. All of the identified outliers were documented separately. RIs were calculated using nonparametric methods if more than 120 reference samples were available or were calculated using robust methods for fewer than 120 reference samples from the adjusted and retested data after outlier removal. For the lower reference limit (LRL), the 2.5th percentile was detected, and for the upper reference limit (URL), the 97.5th percentile was detected. Furthermore, the 90% confidence intervals (CIs) of the LRL and URL were determined. To show the suitability of the new RIs, the widths of the CIs were divided by the widths of the RIs. A ratio of < 0.2 indicated RI accuracy.

The population was partitioned according to physiologic criteria, reproductive status, age, DIM, and sample material used. Subsequently, the partitioning, e.g., S versus LP, pregnant versus nonpregnant, and transit phase, including post‐transit phase (< 42 DIM) versus remaining lactation (≥ 42 DIM), was statistically tested in accordance with Harris and Boyd [13]. Therefore, a subgroup ratio was calculated by dividing the higher SD by the smaller SD. If the subgroup ratio was greater than 1.5, the mean values were compared using the standard normal derivative test. The *z* value was determined using the following formula: *z* = mean_1_ − mean_2_/([SD_1_^2^/*n*_1_] + SD_2_^2^/*n*_2_)^0.5^. Subgroups were considered accurate if the *z* value exceeded the alternative critical *z* statistic. Alternative critical *z* statistic = 3*(*n*_average_/120)^0.5^ [13]. A combined RI was considered sufficient if the subgroup ratio was < 1.5 or if the *z* value was lower than the alternative critical *z* statistic.

All results are presented as the means ± standard deviations or, in the case of non‐normally distributed data, as medians ± average absolute deviations.

## 3 Results

### 3.1 Study Population

Among the 131 lactating Holstein Friesian cows, 62 were pregnant, and 69 were not pregnant. The BCS was 3.0 ± 0.25. The sampled animals were in their first to seventh lactations. The age of the animals was 4.0 ± 1.3 years. On average, the DIM was 198 ± 96 days, and the milk yield was 33.8 ± 9.5 kg/L. All animals met the prespecified inclusion criteria; thus, no animals were excluded before analysis.

### 3.2 Preanalytical Parameters

**FIGURE 1:**
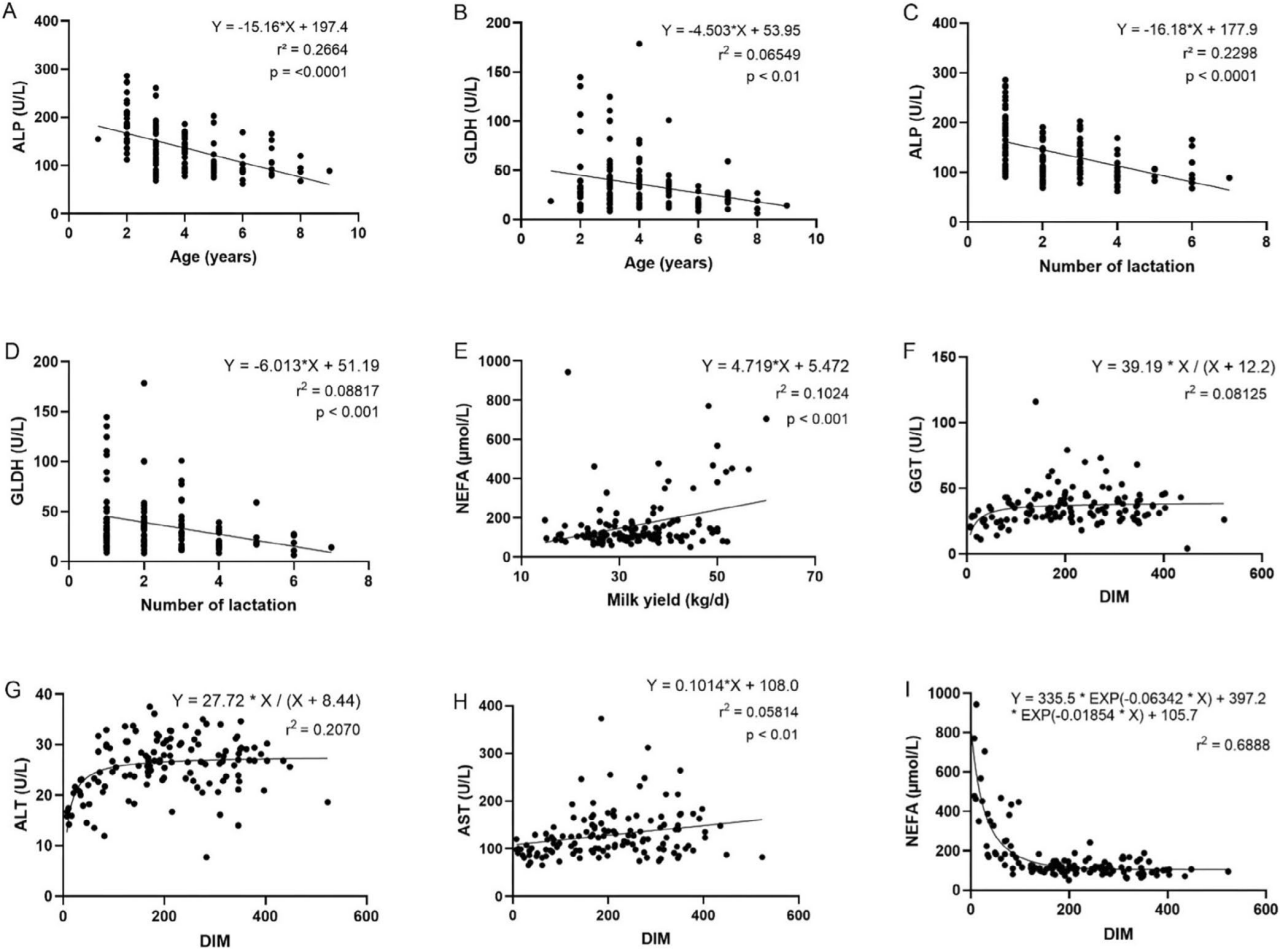
Scatterplots showing the correlation between (A) alkaline phosphatase (ALP) levels and age; (B) glutamate dehydrogenase (GLDH) levels and age; (C) ALP levels and the number of lactations; (D) GLDH levels and the number of lactations; (E) nonesterified fatty acid (NEFA) levels and milk yield; (F) gamma‐glutamyl transferase (GGT) and days in milk (DIM); (G) alanine aminotransferase (ALT) levels and DIM; (H) aspartate aminotransferase (AST) levels and DIM; (I) NEFA and DIM.

**FIGURE 2:**
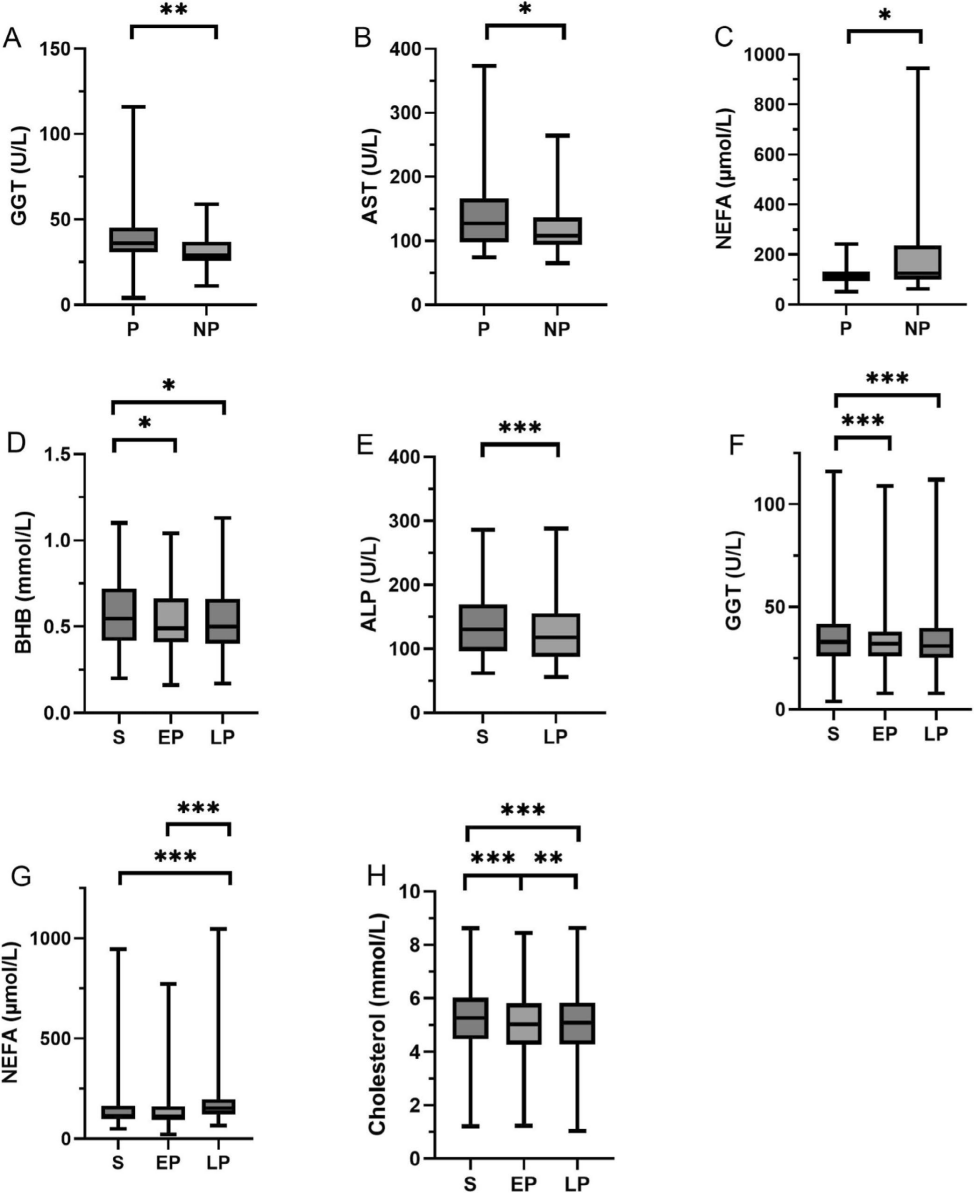
Boxplots showing the comparison between (A) gamma‐glutamyl transferase (GGT) activity in serum from pregnant (P) and nonpregnant (NP) cows; (B) aspartate aminotransferase (AST) activity in serum from P and NP cows; (C) nonesterified fatty acid (NEFA) concentration in serum from P and NP cows; (D) beta‐hydroxybutyrate (BHB) concentration in serum (S), EDTA plasma (EP) and lithium heparin plasma (LP); (E) alkaline phosphatase (ALP) concentration in S and LP; (F) GGT concentration in S, EP, and LP; (G) NEFA concentration in S, EP, and LP; (H) cholesterol concentration in S, EP, and LP. *P < 0.05; **P < 0.01; ***P < 0.001.

A negative correlation between age (*r* = −0.5530) and number of lactations (*r* = −0.5142) was demonstrated for ALP. Furthermore, a negative correlation between age (*r* = −0.2525) and number of lactations (*r* = −0.33545) was demonstrated for GLDH. Reduced ALT and GLDH activities were observed with increasing age and number of lactations. The concentration of NEFAs was positively correlated with milk yield (*r* = 0.2511). No associations were found between the BCS and the concentrations of the variables studied. A positive correlation with DIM was observed for GGT (*r* = 0.2701), ALT (*r* = 0.2411), and AST (*r* = 0.3062), and a negative correlation was observed for NEFA concentration and DIM (*r* = −0.5860). The *p* values of the correlation analysis are listed in Table 3. A regression analysis was carried out for all values for which a correlation could be found. Linear regression was performed for the variable ALP in the serum with age (*r* ^2^ = 0.2664) and number of lactations (*r* ^2^ = 0.2298, Figure 1A,C). No association between age (*r* ^2^ = 0.06549) and number of lactations (*r* ^2^ = 0.08817) was demonstrated for GLDH (Figure 1B,D). The concentration of NEFAs in the serum exhibited a positive linear regression with milk yield (*r* ^2^ = 0.1024, Figure 1E). A positive association with DIM was observed for ALT (*r* ^2^ = 0.2070, Figure 1G), but no associations were detected for GGT (*r* ^2^ = 0.08125, Figure 1F) or AST (*r* ^2^ = 0.05814, Figure 1H). The concentrations of NEFAs in the serum exhibited exponential dissociation with increasing DIM (*r* ^2^ = 0.6888, Figure 1I). The *p* values for the regression analyses are shown in Figure 1. Pregnant cows had increased GGT (pregnant: 36.0 ± 11.5 U/L; nonpregnant: 29.0 ± 6.9 U/L) and AST (pregnant: 127.5 ± 42.9 U/L; nonpregnant: 108.0 ± 26.3 U/L) activities (Figure 2A,B). However, pregnant animals presented lower NEFA concentrations (110.0 ± 25.6 μmol/L) than nonpregnant animals did (126.0 ± 131.0 μmol/L; Figure 2C). A significant correlation of serum, EP, and LP with enzyme activity and metabolite concentrations was demonstrated for beta‐hydroxybutyrate (BHB), ALP, GGT, NEFA, cholesterol, and AST (Figure 2D–H). Higher values in serum than in plasma were measured for BHB (S: 0.6 ± 0.2 mmol/L; EP: 0.5 ± 0.2 mmol/L; LP: 0.5 ± 0.2 mmol/L), ALP (S: 130.0 ± 39.3 U/L; LP: 119.0 ± 36.3 U/L), GGT (S: 33.0 ± 9.6 U/L; EP: 32.0 ± 8.7 U/L; LP: 31.0 ± 9.0 U/L), cholesterol (S: 5.2 ± 1.2 mmol/L; EP: 5.0 ± 1.2 mmol/L; LP: 5.0 ± 1.2 mmol/L), and AST (S: 120.0 ± 35.0 U/L; EP: 116.0 ± 35.8 U/L; LP: 115.0 ± 35.8 U/L). The concentration of NEFAs was higher in LP (154.0 ± 85.8 μmol/L) than in serum (116 ± 85.5 μmol/L) and EP (113.0 ± 83.6 μmol/L). For GLDH (S: 27.5 ± 19.7 U/L; EP: 27.6 ± 19.8 U/L; LP: 28.0 ± 19.0 U/L), CK (S: 121.0 ± 36.7 U/L; EP: 123.0 ± 36.1 U/L; LP: 127.0 ± 36.2 U/L), and ALT (S: 25.7 ± 5.5 U/L; EP: 25.8 ± 5.3 U/L; LP: 25.4 ± 5.4 U/L), no influence of the anticoagulant used was detected. The *p* values of this analysis are listed in Table 3.

**TABLE 3:** Used method and *p* values of the preanalytical influence analysis.

Variable	Age	Number of lactations	Milk yield	BCS	DIM	Pregnancy	Material
BHB	SK, 0.02	SK, 0.20	SK, 0.52	SK, 0.44	SK, 0.52	MW, 0.70	FT, < 0.001
ALP	SK, < 0.0001	SK, < 0.0001	SK, 0.21	SK, 0.07	SK, 0.67	MW, 0.36	MW, < 0.0001
GLDH	SK, < 0.01	SK, < 0.0001	SK, 0.13	SK, 0.42	SK, 0.07	MW, 0.12	FT, 0.81
CK	SK, 0.08	SK, 0.04	SK, 0.44	SK, 0.33	SK, 0.90	MW, 0.35	FT, 0.17
GGT	SK, 0.13	SK, 0.65	SK, 0.83	SK, 0.71	SK, < 0.01	MW, < 0.001	FT, < 0.0001
NEFA	SK, 0.16	SK, 0.79	SK, < 0.01	SK, 0.71	SK, < 0.0001	MW, < 0.01	FT, < 0.0001
ALT	PK, 0.09	PK, 0.01	PK, 0.14	PK, 0.02	PK, < 0.01	UT, 0.06	RA, 0.03
Cholesterol	PK, 0.10	PK, 0.02	PK, 0.05	PK, 0.06	PK, 0.8	UT, 0.09	RA, < 0.0001
AST	SK, 0.16	SK, 0.02	SK, 0.99	SK, 0.39	SK, < 0.001	MW, < 0.01	FT, 0.01
Total bilirubin	SK, 0.77	SK, 0.79	SK, 0.45	SK, 0.97	SK, 0.01	MW, 0.03	ND

Abbreviations: ALP, alkaline phosphatase; ALT, alanine aminotransferase; AST, aspartate aminotransferase; BHB, beta‐hydroxybutyrate; CK, creatine kinase; FT, Friedman test; GGT, gamma‐glutamyltransferase; GLDH, glutamate dehydrogenase; MW, Mann–Whitney *U* test; ND, not determined; NEFA, nonesterified fatty acids; PK, Pearson's correlation; RA, repeated‐measures ANOVA; SK, Spearman's correlation; UT, unpaired *t* test.

### 3.3 Reference Intervals

The established RIs are shown in Table 4.

**TABLE 4:** Reference intervals for standard clinical chemistry analytes in lactating Holstein Friesian cows in Germany.

Measurand	SI units	Initial (*n*)	Removed outliers	Final (*n*)	Mean	SD	Median	Min	Max	Normality test *p‐*value	Symmetry test *p*	Distribution	Method	LRL of RI	URL of RI	0.2 width of RI	CI 90% of LRL	CI 90% of URL
BHB																		
Se*	mmol/L	130	0	130	0.57	0.20	0.54	0.20	1.10	0.45	0.88	G, S	NP, T	0.25	1.00	0.15	0.23–0.26	0.96–1.05
ALP																		
Se*	U/L	130	0	130	137.3	48.7	130.0	62.0	286.0	0.15	0.50	G, S	NP, T	71.1	258.9	37.4	68.0–74.3	245.0–273.8
GLDH																		
Se*	U/L	131	0	131	36.2	29.2	27.5	6.3	178.6	0.87	0.99	G, S	NP, T	9.1	121.0	22.4	8.5–9.8	106.5–138.0
CK																		
Se*	U/L	131	0	131	135.7	48.5	121.0	51.0	293.0	0.08	0.67	G, S	NP, T	65.4	257.2	38.4	63.0–68.0	243.4–272.1
GGT																		
Pregnant, Se*	U/L	62	1	61	39.0	13.7	36.0	4.0	79.0	< 0.01	0.30	NG, S	R, T	8.7	65.3	11.3	3.4–14.6	58.5–71.0
Not pregnant, Se*	U/L	69	0	69	31.0	8.8	29.0	11.0	59.0	0.33	0.39	G, S	R	12.3	48.6	7.3	9.2–15.9	44.6–51.9
NEFA																		
> 42 DIM, Se*	μmol/L	118	0	118	131.8	71.3	112.5	51.0	468.0	0.19	0.38	G, S	R, T	65.4	308.7	48.7	61.7–69.8	249.4–395.1
ALT																		
Se*	U/L	130	0	130	25.7	5.5	26.5	7.7	37.5	0.06	0.34	G, S	NP	14.0	34.5	4.1	13.3–14.8	33.7–35.3
Cholesterol																		
Se*	mmol/L	131	0	131	5.2	1.0	5.3	1.2	8.6	0.14	0.52	G, S	NP	2.7	7.5	1.0	2.5–2.8	7.3–7.6
AST																		
Pregnant, Se*	U/L	62	0	62	142.1	58.5	127.5	74.0	373.0	0.69	0.98	G, S	R, T	76.6	279.7	40.6	73.8–79.5	256.5–306.7
Not pregnant, Se*	U/L	69	1	68	114.5	29.3	107.5	65.0	193.0	0.57	0.91	G, S	R, T	67.1	187.1	24.0	62.1–72.4	171.8–205.6
Total bilirubin																		
Se	μmol/L	131	0	131	2.8	1.0	2.7	0.3	5.4	0.37	0.69	G, S	NP, T	0.9	5.2	0.8	0.8–1.0	5.0–5.3

*Note:* Test used for normality assessment: Shapiro–Wilk test (*p* ≥ 0.05); symmetry test: Kolmogorov–Smirnov test (*p* ≥ 0.05).

Abbreviations: ALP, alkaline phosphatase; ALT, alanine aminotransferase; AST, aspartate aminotransferase; BHB, beta‐hydroxybutyrate; CK, creatine kinase; DIM, days in milk; G, Gaussian; GGT, gamma‐glutamyltransferase; GLDH, glutamate dehydrogenase; LP, lithium heparin plasma; NEFA, nonesterified fatty acids; NG, non‐Gaussian; NP, nonparametric; R, robust; S, symmetric; Se, serum; Se*, reference interval for serum and lithium heparin plasma; T, transformed.

## 4 Discussion

The aim of this study was to determine and establish RIs in association with preanalytical factors in adult lactating Holstein Friesian cows. The newly established RIs may help ensure more precise monitoring and interpretation of laboratory results for dairy herd health management in Germany. The RIs from previous studies for BHB, CK, ALT, cholesterol, and total bilirubin were comparable to the results of this study, despite geographical differences [6, 7, 8]. The URL of ALP was up to 100 U/L above the URL of ALP in comparable studies [6, 8]. The influence of the sample material on the measured values has not yet been investigated for ALP activity in samples from dairy cattle, but the results appear comparable to those from studies on buffalo [15]. Interestingly, no influence of lactation or parity on the ALP concentration, as was shown in previous studies, was demonstrated in the present study [7, 16]. No recent RI studies on GLDH activity could be found for adult female Holstein Friesian dairy cattle in lactation. Older sources give an RI of < 30 U/L. [17] This value is below the URL of the present study (primiparous cows: 127 U/L, pluriparous cows: 134 U/L). This could be due to different measurement techniques or different housing and feeding conditions of the cattle, as well as the progressive breeding of high‐yielding dairy cows and geographical influences [5]. The difference in parity was only partially reflected in the RI. The difference between the URLs was only 8 U/L. From this, it can be concluded that the RIs are sufficient for primiparous and pluriparous animals. Similar RI studies reported comparable values for GGT [6]. However, other studies have shown lower RIs (18.9–20.6 U/L; < 16 U/L) [7, 18]. The observation of higher GGT activity in pregnant cows is consistent with the results of Antanaitis et al. and Stojević et al. [19, 20] Interestingly, this does not seem to be a phenomenon of all animal species; mares, in comparison, show a slight reduction in GGT activity during pregnancy [21]. Due to a slight difference of 12 U/L between the material‐dependent RIs and the material‐independent RI, the latter can be considered sufficient for evaluating GGT activity clinically. The highest NEFA concentrations in dairy cows are measured in the first week postpartum [1, 22]. Similar RIs for NEFAs were reported in previous studies [1, 8]. The decrease in NEFA concentrations over the course of lactation was also considered. In one study, lower NEFA RIs were reported [7]. However, the differences compared with our study can be explained by the fact that only animals in late pregnancy and in the dry period were sampled, which were excluded from the present study. Additionally, higher NEFA concentrations in the heparin plasma of cattle have been reported in previous studies [23, 24]. In those two studies, the use of EDTA as an anticoagulant was recommended. The adapted RI protocol for the sample material established in the present study allows the practicing veterinarian to freely choose the sample tube, especially for NEFAs, thus increasing flexibility while ensuring preanalytical quality. A positive correlation between milk yield and NEFA concentration due to increased fat mobilization, which is associated with high milk production, has also been reported in previous studies [1, 25]. Taking this influence into account enables more precise monitoring and early prophylactic measures to be taken in the event of an early metabolic imbalance in cows in a herd with different stages of production. Further studies are needed to establish RIs for the first weeks postpartum so that monitoring can be as rigorous as necessary during the critical phase of adaptation. The URLs for AST activity reported in the literature are well over 50% lower than those found in the present study [7, 8]. This may be due to the different selected populations, geographical influences, or different sample handling and measurement methods [7, 8]. Cozzi et al. observed a decrease in AST activity over the course of lactation but did not consider the relationship with pregnancy [8]. The fact that pregnant cows have higher AST activities has been shown in other studies, as has the influence of parity [1, 20]. The effect of neither pregnancy nor parity could be confirmed in the present study.

One of the limitations of this study is the health status of the animals. A detailed general examination was carried out by one veterinarian, but no further tests, such as one to evaluate the acute phase protein haptoglobin level, were performed. There remains a risk that animals with subclinical disease were included in the study. The determination of haptoglobin levels would have enabled the identification of inapparent infections in some animals. A further limitation is the photometric analysis. All test kits used are intended for commercial use in human medicine and have been validated for this purpose by the manufacturers. Despite internal laboratory validation and testing with human and specially produced bovine controls, measurement inaccuracies may occur in cross‐species use. Furthermore, for some variables, including GLDH, NEFAs, and AST, the 90% CIs were more than 0.2 times wider than the RIs. This may be an indication that the sample size was too small [2]. Further RI studies are needed for the analytes mentioned. Notably, the AST activity results should also be interpreted with caution. From approximately 100 DIM, an increase in the variance of the values was observed in this study. A higher AST activity may be of muscular origin, which can be caused by stress, unrest, and subsequent trauma within the herds [1, 20]. In this case, a parallel increase in CK activity would be expected. The altered AST levels may also be of hepatic origin. Further studies are needed to clarify whether such high AST activities in dairy cows are of physiological or pathological origin. In conclusion, the RIs calculated in this study are more accurate than those in previous studies, according to the current state of scientific knowledge and considering preanalytical factors. Therefore, the RIs can be safely used to classify laboratory results for Holstein Friesian cows during lactation.

## Conflicts of Interest

The authors declare no conflicts of interest.
